# Pectin-Lipid Self-Assembly: Influence on the Formation of Polyhydroxy Fatty Acids Nanoparticles

**DOI:** 10.1371/journal.pone.0124639

**Published:** 2015-04-27

**Authors:** Susana Guzman-Puyol, José Jesús Benítez, Eva Domínguez, Ilker Sefik Bayer, Roberto Cingolani, Athanassia Athanassiou, Antonio Heredia, José Alejandro Heredia-Guerrero

**Affiliations:** 1 Smart Materials, Nanophysics, Fondazione Istituto Italiano di Tecnologia (IIT), via Morego 30, 16163, Genoa, Italy; 2 Instituto de Ciencia de Materiales de Sevilla (ICMS), Centro mixto CSIC-Universidad de Sevilla, Avda. Americo Vespuccio 49, Isla de la Cartuja, E-41092, Sevilla, Spain; 3 Instituto de Hortofruticultura Subtropical y Mediterránea (IHSM) La Mayora, Universidad de Málaga-CSIC, Algarrobo-Costa, E-29750, Málaga, Spain; 4 Fondazione Istituto Italiano di Tecnologia (IIT), via Morego 30, 16163, Genoa, Italy; 5 Departamento de Biología Molecular y Bioquímica, IHSM La Mayora, UMA-CSIC, Universidad de Málaga, E-29071, Málaga, Spain; George Mason University, UNITED STATES

## Abstract

Nanoparticles, named *cutinsomes*, have been prepared from aleuritic (9,10,16-trihidroxipalmitic) acid and tomato fruit cutin monomers (a mixture of mainly 9(10),16-dihydroxypalmitic acid (85%, w/w) and 16-hydroxyhexadecanoic acid (7.5%, w/w)) with pectin in aqueous solution. The process of formation of the nanoparticles of aleuritic acid plus pectin has been monitored by UV-Vis spectrophotometry, while their chemical and morphological characterization was analyzed by ATR-FTIR, TEM, and non-contact AFM. The structure of these nanoparticles can be described as a lipid core with a pectin shell. Pectin facilitated the formation of nanoparticles, by inducing their aggregation in branched chains and favoring the condensation between lipid monomers. Also, pectin determined the self-assembly of *cutinsomes* on highly ordered pyrolytic graphite (HOPG) surfaces, causing their opening and forming interconnected structures. In the case of cutin monomers, the nanoparticles are fused, and the condensation of the hydroxy fatty acids is strongly affected by the presence of the polysaccharide. The interaction of pectin with polyhydroxylated fatty acids could be related to an initial step in the formation of the plant biopolyester cutin.

## Introduction

The cuticle is a composite membrane that covers the epidermis of non-lignified aerial parts of plants [1]. It is an effective barrier against massive water loss, pathogen and fungal infection and UV radiation. It also prevents organ fusion, provides mechanical support and, in some cases, self-cleaning surfaces [2,3]. The plant cuticle is composed by an amorphous and insoluble matrix named cutin, polysaccharides from the cell wall (mainly cellulose, hemicellulose and pectin) and other components such as waxes and phenolic compounds [4]. Cutin is an aliphatic polyester with interesting intrinsic properties (hydrophobicity, viscoelasticity, chemical inertness, etc.) formed by condensed C_16_ or C_18_ polyhydroxy fatty acids [4]. Currently, there is a growing interest in this biopolymer due to mainly two reasons. First, cutin is a key factor in the quality and appearance of commercial fruits [5,6]. Second, as consequence of its abundance in nature and of its above-mentioned properties, it is starting to be considered a source of lipid monomers as well as a model to produce new bio-inspired materials [7–10].

In recent years, a large number of publications have addressed cutin biosynthesis. Practically all of them were focused on the identification of genes and enzymes involved in the synthesis of this polyester [2,3,11,12]. In fact, a cutin synthase, specifically a tomato extracellular acyltransferase, was identified as the enzyme mainly responsible for cutin synthesis [13]. However, other mechanisms have been suggested to be involved in this process. In this sense, a nanometric approach for cutin biosynthesis has been proposed [14,15]. This hypothesis takes into account the self-assembly properties of cutin polyhydroxylated fatty acids in polar environments and their ability to produce self-esterification reactions at neutral pH [14]. Particles thus synthetized, *cutinsomes*, have been described as soft sphere-like structures (50–200 nm diameter) with a mostly esterified liquid-like core surrounded by an acid carboxylic/carboxylate shell [16]. *Cutinsomes* have shown a high capacity of aggregation, forming micro-islands on cellulose and cuticle substrates with similar characteristics to natural cutin [15,17]. Participation of *cutinsomes* in cutin biosynthesis has been demonstrated after their detection in the epidermis of different plant species [12,18,19]. The complementarity of this nanometric approach, where the *cutinsomes* are extruded and aggregated during the initial stages of the cutin formation, with the proposed enzymatic mechanism has been suggested [12].

To our knowledge, the role of polysaccharide, the other main cuticle component, in cutin biosynthesis has not been addressed. Here we report the influence of low amounts of pectin on the self-assembly in an aqueous environment of aleuritic acid and tomato cutin monomers. Pectin is one of the most important polysaccharide present in the plant cell wall and in the cuticle, and therefore its putative involvement in *cutinsome* formation has been studied.

## Materials and Methods

### Materials

Aleuritic (DL-*threo*-9,10,16-trihydroxyhexadecanoic, C_16_H_32_O_5_) acid (93.8% by NaOH titration) was purchased from Fluka. Apple pectin (≥ 76% of galacturonic acid, ˜7% of methoxi group content) was purchased from Sigma-Aldrich. Both materials were used without additional treatments.

### Extraction of cutin monomers

Previously to the extraction of cutin monomers, tomato (*Solanum lycopersicum* L.) fruit cuticles were enzymatically isolated in an aqueous solution of sodium citrate buffer (50 mM, pH 3.7) containing fungal cellulase (0.2%, w/v, Sigma, St Louis, MO, USA) and pectinase (2.0%, w/v, Sigma) with 1mM NaN_3_ to prevent microbial growth [20]. Vacuum was applied to facilitate enzyme penetration and fruits were incubated with continuous agitation at 37°C for 10–14 days. Cuticles were then separated from the epidermis, rinsed in distilled water, and stored under dry conditions.

Cutin monomers were extracted following a protocol similar to that described by Luque *et al*. [21]. First, cuticle waxes were removed after incubation in a chloroform:methanol mixture (2:1, v/v) for 3 h at approximately 65°C. Cutin was obtained after hydrolysis of the polysaccharide fraction of dewaxed cuticles in a 6 M HCl solution for 12 h at 105°C. Finally, cutin was depolymerized in a KOH 2% (w/v) solution for 18 h at 100°C. Cutin monomers were obtained by extraction in diethyl ether and evaporation of the organic solvent under N_2_.

### Nanoparticle preparation

Nanoparticles of polyhydroxy fatty acids and pectin were produced using a method previously described [22]. Basically, nanoparticles were prepared by adding aleuritic acid (30 mg/mL) or cutin monomers (3 mg/mL) to alkaline (NaOH 0.5 M) solutions of pectin (0, 1.25, 2.5, and 5 mg/mL for aleuritic acid and 0 and 0.125 mg/mL for cutin monomers) at pH 12 and reducing the pH to 5.8 with small amounts of HCl 0.1 M. Thus, final fatty acid:pectin weight ratio was 1:0, 24:1, 12:1, 6:1 for aleuritic acid:pectin and 1:0, 6:1 for cutin monomer:pectin mixtures. Nanoparticles were obtained after centrifugation at 19000 *g*. They were then washed several times with distilled water and filtered to remove residual fatty acids and pectin and sodium chloride formed during the process. Dispersions were prepared using 1.5 mg of nanoparticles in 1.5 mL of distilled water and sonicated for 1 h to break the aggregates formed after centrifugation.

### Nanoparticle characterization

Opalescence of the solutions was monitored at 480 nm by UV-Vis spectroscopy using a SmartSpec Plus (Bio-Rad) spectrophotometer. Four mL cells with 10 mm path length were employed. Changes in absorbance were monitored as the pH of the different fatty acid:pectin solutions was decreased from 12 to ˜ 5.8 with small amounts of HCl 0.1 M (at pH < 5.8 fatty acids precipitated). Also, opalescence of pectin solutions (2.5 and 5 mg/mL) at pH 12 was measured.

For Transmission Electron Microscopy (TEM) analysis, a drop of nanoparticles dispersed in water was deposited on a copper grid and allowed to dry. Nanoparticles were stained with an aqueous solution of uranyl acetate (1%, w/v) to contrast lipid material, rinsed with distilled water and then analyzed at 100 kV with a Philips CM-100 electron microscope. Particle dimensions were measured using ImageJ software analysis.

Attenuated Total Reflected-Fourier Transform Infrared (ATR-FTIR) spectra of samples were obtained from pellets of purified nanoparticles using an ATR accessory (MIRacle ATR, PIKE Technologies) coupled to a FTIR spectrometer (FT/IR-4100, JASCO). All spectra were recorded in the 4000–600 cm^-1^ spectral range at 4 cm^-1^ of resolution and accumulating 128 scans.

For Atomic Force Microscopy (AFM) measurements, a droplet (25 μL) of disperse nanoparticles in water was deposited on a freshly cleaved surface of highly ordered pyrolytic graphite (HOPG) and slowly evaporated by keeping the sample at 6°C for 24h. The AFM was a Cervantes model from Nanotec Electrónica operated at room conditions (20–25°C and 35–40% RH). Non-contact images were obtained and processed using the WSxM software [23]. Rectangular Si_3_N_4_ cantilevers with nominal force constant of 2.8 N m^-1^ and resonance frequency around 80 kHz were used.

## Results and Discussion

### Characterization of pectin-aleuritic acid nanoparticles

**Fig 1:**
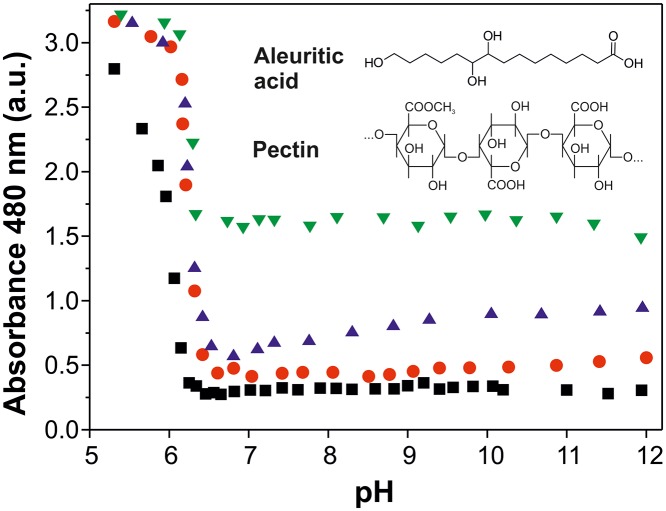
Formation of *cutinsomes* with pectin monitored by opalescence. Transition of aleuritic acid (black squares) and aleuritic acid with pectin (24:1 w/w-red circles, 12:1 w/w-blue triangles, 6:1 w/w-green inverted triangles) solutions from micellar to nanoparticle state monitored at 480 nm. The absorbance increase is associated with the opalescence resulting from the appearance and aggregation of nanoparticles. Chemical structures of pectin and aleuritic acid are included.

Presence of nanoparticles was monitored by the titration curves of the solutions of aleuritic acid and aleuritic acid:pectin mixtures at different ratios (24:1, 12:1, and 6:1, w/w) and the measurement of opalescence, Fig 1. The sample of aleuritic acid without pectin showed low values of absorbance at basic pH with a significant increase at lightly acid pH (˜ 6.3). This behavior has been described for other fatty acid molecules and can be related to a direct transition from micelles to nanodrops [22]. The 24:1 (w/w) sample of aleuritic acid:pectin was very similar, but the turning point was shifted to pH 6.6. This small increase in pH (0.3 points) observed in the sample containing pectin can be biologically relevant and suggests pectin participation in the formation of nanoparticles. Samples with higher ratios of pectin were comparable to that of 24:1 ratio (aleuritic:pectin), but with higher absorbance values in the 12–6.6 pH range. This increase in absorbance was also observed in the samples of pectin without aleuritic acid: ˜ 0.7 and ˜ 1.5 a. u. in the 2.5 and 5 mg/mL pectin solutions, respectively. These results suggest that pectin increased light scattering in this wavelength region.

**Fig 2:**
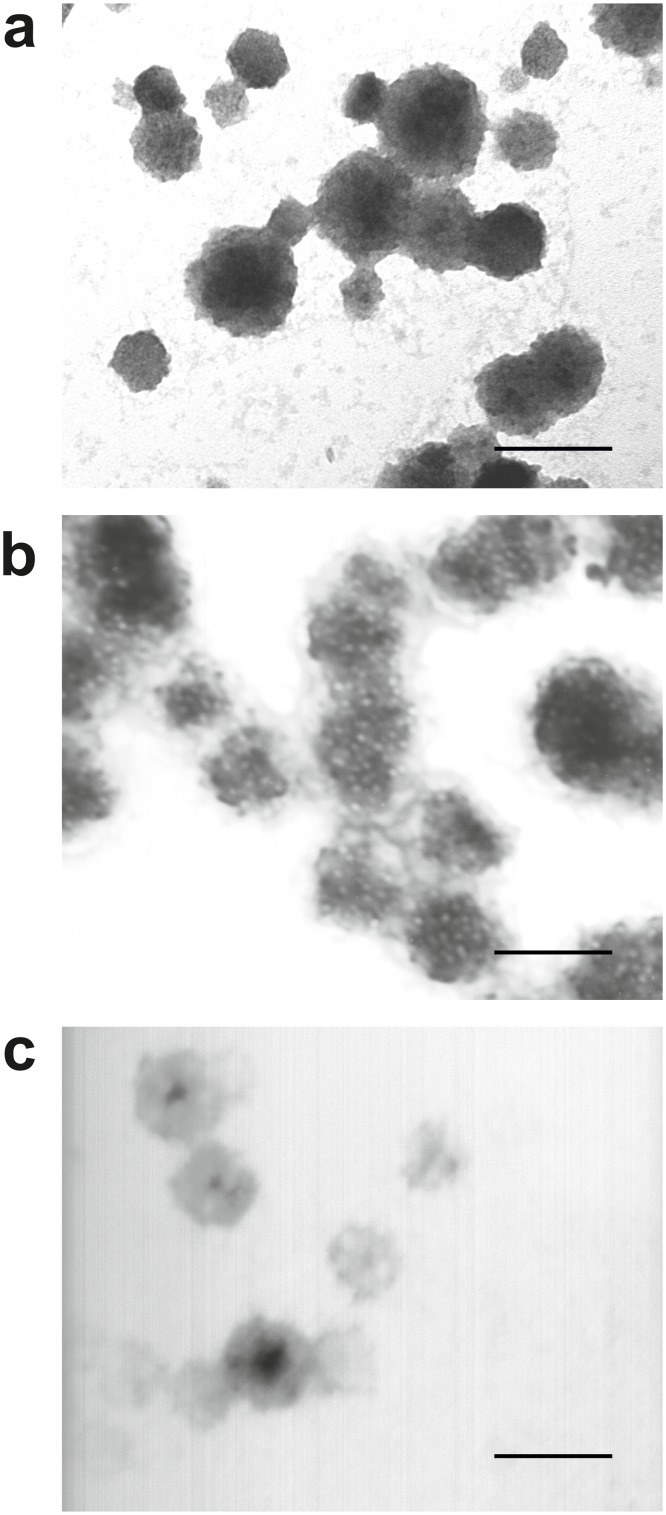
Morphology of *cutinsomes* from aleuritic acid and pectin. **a-c**, TEM images of aggregated nanoparticles of aleuritic acid and pectin at 1:0 (a), 24:1 (b) and 6:1 (c) w/w ratios. Diverse morphologies are observed for the different ratios. Scale bar = 200 nm.

Morphology of the structures formed from mixtures of aleuritic acid and pectin was characterized by TEM, Fig 2, using uranyl acetate to stain aleuritic acid since this compound has high affinity for lipid molecules However, uranyl acetate lightly stained pectin, S1 Fig [24]. Fig 2a shows a TEM micrograph of nanoparticles derived from aleuritic acid without pectin. These *cutinsomes* had a polydisperse size distribution (50–200 nm diameter) and were found either isolated, aggregated or fused [14,16]. Morphology of the nanoparticles obtained with a 24:1 ratio of aleuritic:pectin is shown in Fig 2b. In this case sphere-like nanoparticles with a diameter around 165 nm were observed. These particles displayed an electron dense lipid core with a diameter of 138 ± 15 nm surrounded by an electron translucent pectin shell with a thickness of 26 ± 4 nm. Some unstained regions with a diameter of 9 ± 1 nm were observed in the lipid core which could be related to areas with a higher local concentration of pectin. Many particles formed aggregates joined by their polysaccharide shells, forming branched chains of nanoparticles Fusion of lipid cores was also observed but limited by the encapsulation action exerted by the pectin shell. Finally, Fig 2c shows the nanoparticles derived from the 6:1 ratio of aleuritic:pectin. In this case, a thick pectin shell (56 ± 7 nm) surrounded a much thinner lipid core (28 ± 5 nm). Aggregation was low with most nanoparticles isolated. These data suggest that pectin acts as an effective encapsulating agent of cutin material, providing an interphase between the polar medium and the hydrophobic core of the nanoparticles and regulating the final size and shape of *cutinsomes*.

**Fig 3:**
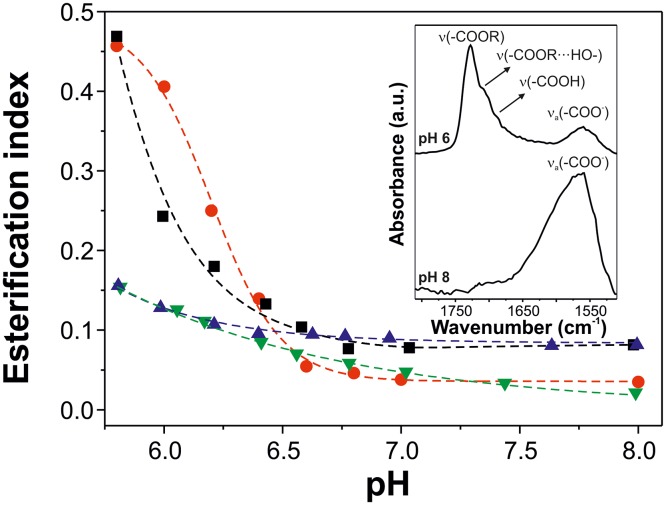
Esterification capacity of *cutinsomes* from aleuritic acid and pectin. Esterification index of the precipitated solids obtained from aleuritic acid (black squares) and aleuritic acid with pectin (24:1 w/w-red circles, 12:1 w/w-blue triangles, 6:1 w/w-green inverted triangles) solutions in the range of pH from 8.0 to 5.6. Inset shows the ATR-FTIR spectra in the carbonyl region (1810–1510 cm^-1^) of the precipitated solid from aleuritic acid with pectin (24:1 w/w) solutions at pH 8 and 6.

*Cutinsomes* have been reported to be formed by partially esterified material [14,15]. Esterification level of the nanoparticles derived from the different aleuritic acid:pectin mixture ratios were evaluated by ATR-FTIR. For this, the esterification index, that is the ratio between the intensities of stretching bands of ester and methylene groups, was calculated at different pH values, Fig 3. This parameter has been used in the chemical characterization of cutin to estimate the presence of ester functional groups [25]. Samples of aleuritic acid and of the 24:1 mixture of aleuritic:pectin showed a similar behavior to that observed in the titration process (see Fig 1), with low values at basic pH and a sudden increase of the esterification index at lightly acid pH. In fact, the turning point of the opalescence and the esterification index was observed at the same pH (˜6.3 for aleuritic acid and ˜ 6.6 for the 24:1 mixture of aleuritic:pectin), indicating that in the micellar state (basic conditions) no reaction occurred while at the nanoparticle state (neutral-lightly acid pH) monomer condensation took place. It is interesting to note that in the 5.8–6.3 pH range, where nanoparticles are formed, values of the esterification index were higher for the 24:1 mixture of aleuritic:pectin than for the aleuritic acid alone. On the other hand, the 12:1 and 6:1 mixtures of aleuritic:pectin showed low esterification values in the pH range studied. Though, a small increase was observed at pH ˜ 6.5. Molecular orientations of—OH and—COOH groups to allow their interaction and extrusion of water molecules generated during monomer condensation have been considered the driving forces of the esterification reaction [14]. Pectin incorporation to the fatty acid mixture produced changes in the esterification reaction of nanoparticles. For instance, the pectin shell, as interphase between the hydrophobic lipid core and the aqueous environment, could regulate the flow of water molecules and shift the reaction to hydrolysis. This effect would be stronger with thicker shells (*e*.*g*., 12:1 and 6:1 mixtures of aleuritic:pectin). Also, reaction between pectin and lipid molecules should be considered. However, further chemical analyses (for example, with nuclear magnetic resonance) would be necessary to confirm these assumptions.

Main chemical modifications occurring in the fatty acid monomers during the formation of *cutinsomes* in the presence of pectin have been characterized by ATR-FTIR. Inset in Fig 3 shows the infrared spectra in the C = O region (1810–1510 cm^-1^) of the 24:1 mixture of aleuritic:pectin at pH 8 and 6. At pH 8, in the micellar regime, a single and strong band at 1562 cm^-1^, ascribed to the asymmetrical stretching of carboxylate functional groups of aleuritic acid and pectin, was detected. At pH 6, after nanoparticles have been formed and monomers reacted, the intensity of this vibration was greatly reduced while a major band at 1728 cm^-1^ associated with the stretching of C = O groups in ester bonds was detected. Also, two shoulders at 1711 and 1687 cm^-1^ were observed and assigned to the C = O stretching of ester groups interacting by hydrogen bonds and carboxylic acids, respectively.

**Fig 4:**
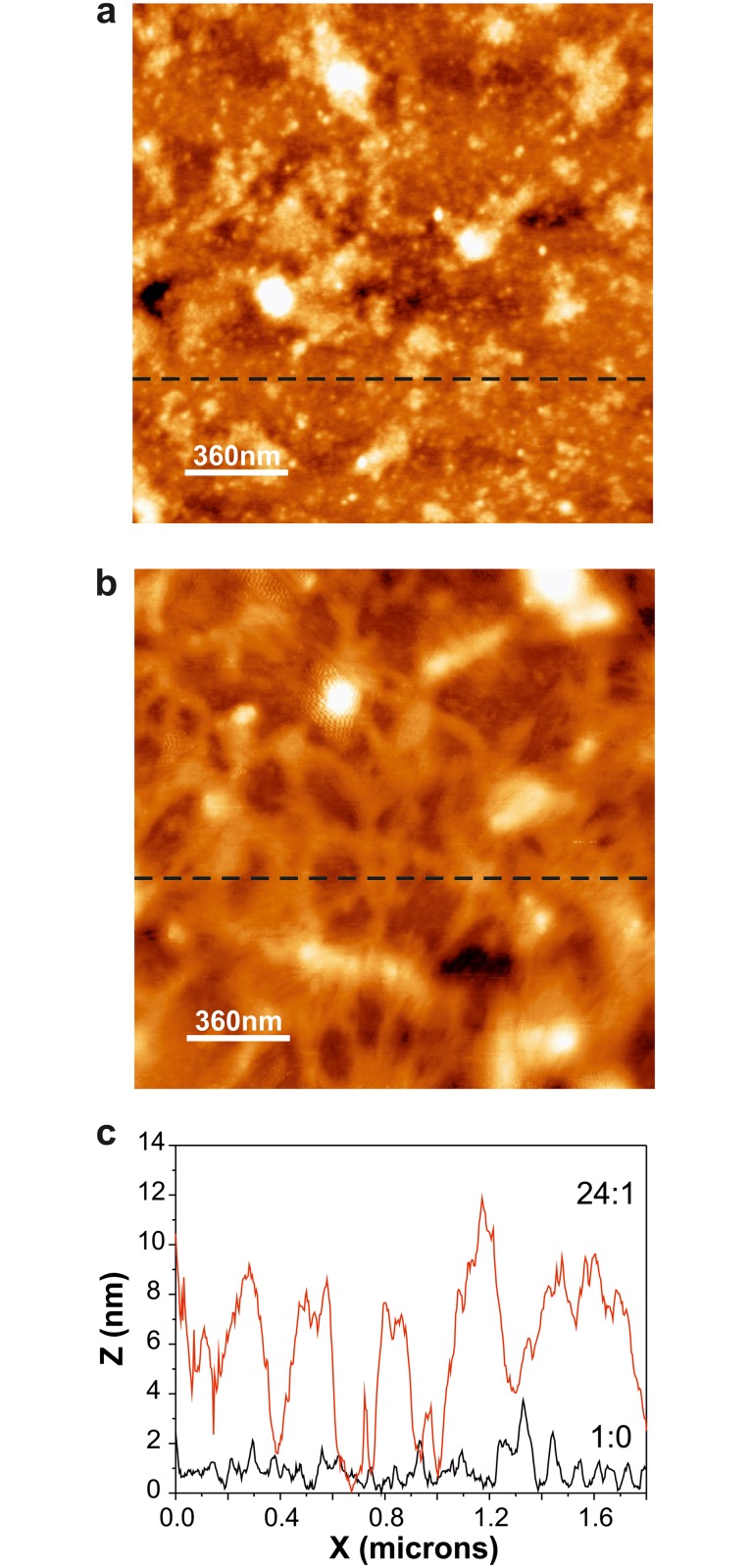
Interaction HOPG surface-*cutinsomes* with pectin. **a-b**, non-contact AFM topography of cutinsomes obtained from mixtures of aleuritic acid with pectin at 1:0 (a) and 24:1 (b) ratios deposited on HOPG by drop vaporization. **c**, height profile corresponding to the dashed line in **a** and **b**.

Structural stability of *cutinsomes* derived from aleuritic acid and the 24:1 mixture of aleuritic:pectin was studied by non-contact AFM after particle deposition on a non-polar substrate (HOPG). *Cutinsomes* obtained from aleuritic acid preserved their structure and aggregated, Fig 4a. This result is very similar to that described for *cutinsomes* derived from cutin monomers on the same substrate [16]. Particles from the 24:1 mixture of aleuritic:pectin showed a structure of interconnected islands with angular hollows, Fig 4b. Moreover, the heights of the structures described were very different: 1–2 nm for aleuritic acid and approximately 8 nm for the 24:1 mixture, Fig 4c. These differences can be ascribed to pectin incorporation to the self-assembly process. It is known that self-assembled polysaccharides on HOPG produce geometric structures due to strong interaction between the C-H functional groups of the polysaccharide with the sp^2^ hybridized orbital of the aromatic rings of the hexagonal graphite crystal geometry [26,27]. In this sense, pectin can participate in the self-assembly of *cutinsomes* inducing the opening of nanoparticles and favoring certain orientations on the HOPG surface during the process.

### Nanoparticles of cutin monomers and pectin

**Fig 5:**
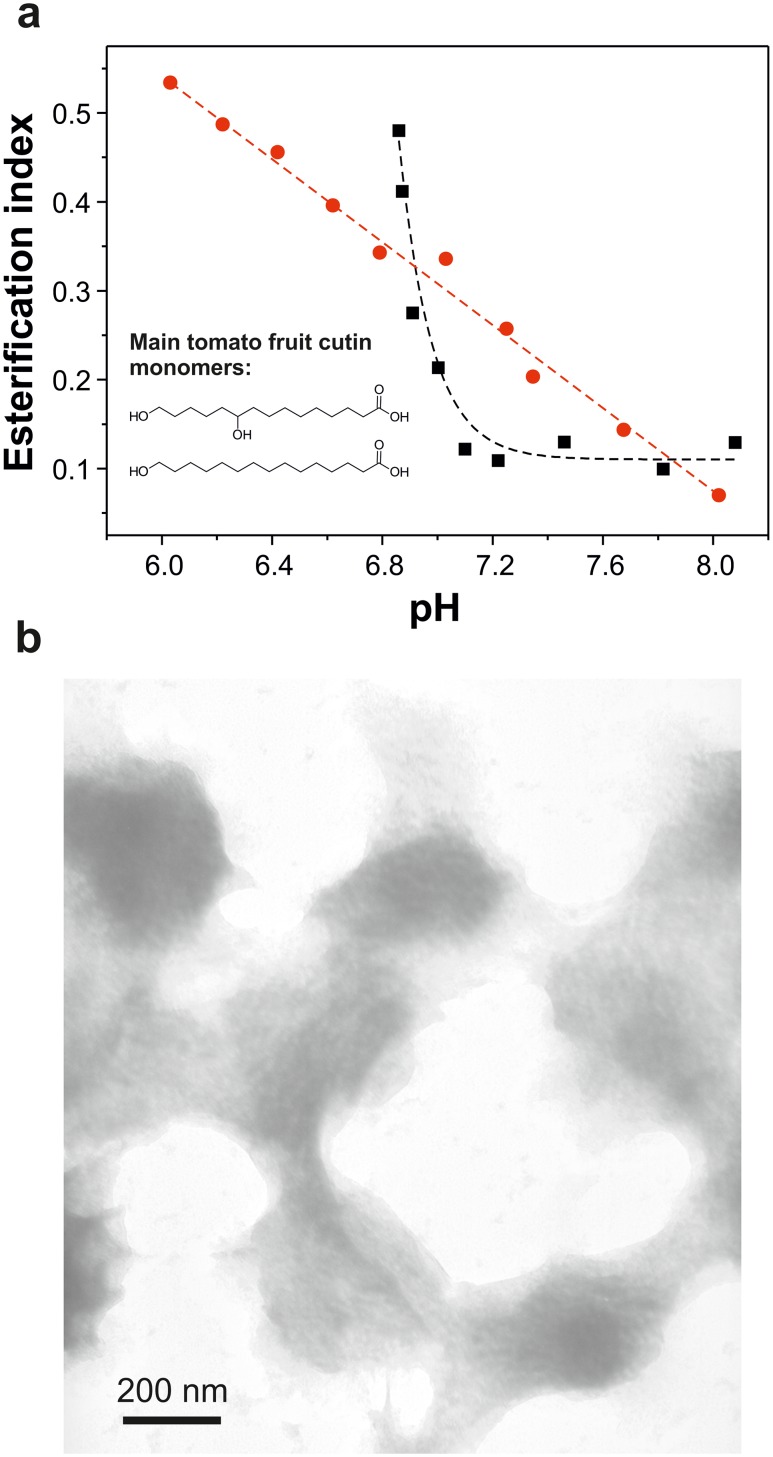
*Cutinsomes* from cutin monomers and pectin. **a**, esterification index of precipitated solids from solutions of cutin monomers (black squares) and cutin monomers with pectin (24:1 w/w-red circles) in the range of pH from 8.5 to 5.5. Chemical structures of the main tomato fruit cutin monomers of tomato fruit are included. **b**, TEM image of aggregated nanoparticles of cutin monomer and pectin at 24:1 w/w. A diffuse edge between the components is observed.

The effect of a low amount of pectin in the formation of nanoparticles from tomato cutin monomers was also studied. Tomato fruit cutin is mainly composed of a mixture of 9(10),16-dihydroxypalmitic acid (85%, w/w) and 16-hydroxyhexadecanoic acid (7.5%, w/w)) [28]. Fig 5a shows the esterification index of cutin monomers and a 24:1 mixture of cutin:pectin. The sample of cutin monomers without pectin showed a similar esterification index as that of aleuritic acid (see Fig 3): low values at basic conditions and a strong increase at neutral conditions. The highest esterification index was achieved at pH ˜ 6.85, below this value hydroxylated fatty acids precipitated. The main difference between the cutin monomer sample and the aleuritic acid one was the shift of the turning point to neutral (˜ 7.1). Samples of 24:1 mixture of cutin:pectin showed that esterification changed linearly with pH, with a maximum achieved at pH ˜ 6.1. To better understand this behavior, nanoparticle structure was analyzed by TEM, Fig 5b. In this case, fused rounded particles with a diameter of 200–300 nm were observed. Most of them showed lipid cores with diffuse edges surrounded by a grey material. Unlike the sample derived from the 24:1 mixture of aleuritic acid:pectin, the polysaccharide and lipid material did not separate but were partially blended.

### A model for the formation of *cutinsomes* with pectin

**Fig 6:**
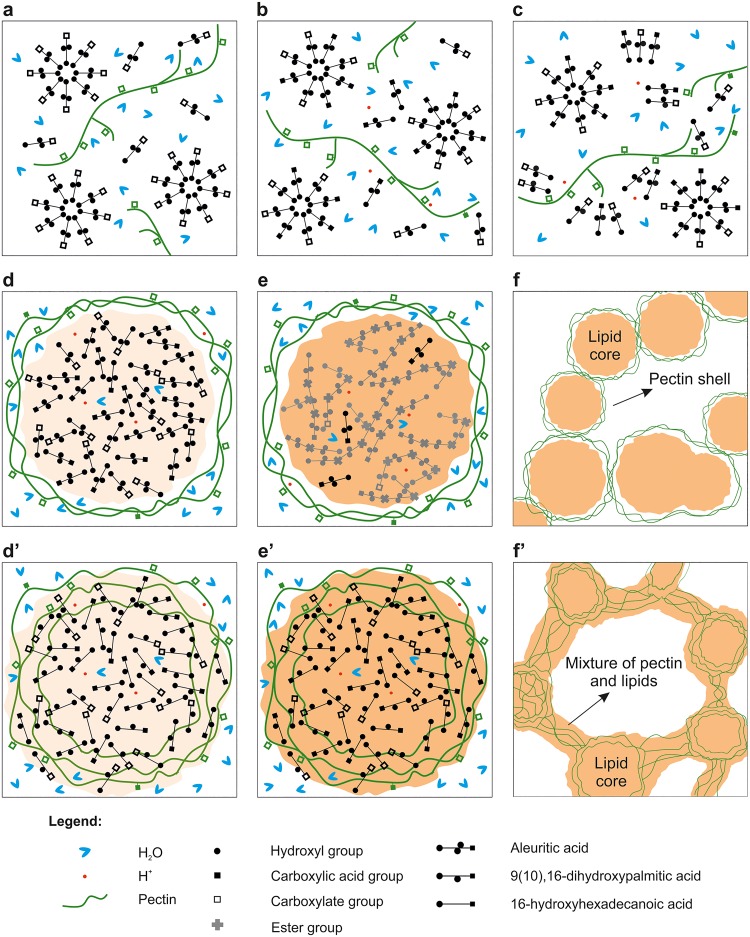
A model for pectin-lipid self-assembly during the formation of *cutinsomes*. Diagram of the phase transition from a micellar state to the formation and polymerization of a *cutinsome* and its subsequent aggregation from an aqueous solution of aleuritic acid (a-f) or cutin monomers (a-c and d’-f’) with pectin.

Based on the above experimental data and by analogy with lipid nanoparticles derived from polyhydroxylated fatty acids [14–18], a model for the formation of *cutinsomes* with pectin is proposed, Fig 6. Fig 6a–6c show the stages that are common to aleuritic acid and cutin monomers, while Fig 6d–6f those specific to aleuritic acid and Fig 6d’–6f’ to cutin monomers. The addition of low relative amounts of pectin has also been considered. At the initial stage, Fig 6a, lipid molecules and pectin are solved in a basic aqueous solution. The carboxylic acid groups of both substances are in the carboxylate form. Lipid molecules are free in the solution or, most probably, forming micelles. At lightly acid pH a critical ratio of fatty acid—COOH and—COO^-^ groups is generated, while pectin, due to its lower pK_a_ continues in the carboxylate form, Fig 6b. At this point, formation of RCOO^-^(RCOOH)_n_ aggregates is favored by hydrogen bond stabilization [23]. These structures can result from the combination of lipid molecules or lipid molecules with pectin chains, Fig 6c. At this point differences between aleuritic acid and cutin monomers start. In the case of the aleuritic acid, when the solution is slightly more acid, these aggregates can self-assemble producing nanoparticles with a polar shell of pectin acting as an interphase between the aqueous medium and a hydrophobic core mainly composed of insoluble aleuritic acid molecules in the—COOH form. Some protons, carboxylated lipids, water molecules, and pectin chains can also be found in the lipid core, Fig 6d. Under these conditions, the right molecular orientation of—OH and—COOH lipid functional groups and the extrusion of water molecules to the environment would allow monomer condensation and generate a polymerized core, Fig 6e. These nanoparticles can aggregate by their pectin shells forming branched chains of esterified polyhydroxy fatty acids. On the other hand, cutin monomers would interact with the polysaccharide originating nanoparticles with lipid cores and blended and diffuse shells, Fig 6d’. These structures gradually esterify as the pH decreases, Fig 6e’. Finally, nanoparticles aggregate forming rounded structures with lipid cores partially mixed with pectin.

## Conclusions

Nanoparticles, named *cutinsomes*, have been prepared from different mixtures of aleuritic acid or cutin monomers with pectin. In the case of aleuritic acid, TEM analysis has revealed that nanoparticles have a lipid core surrounded by a pectin shell. The role of pectin in this system is multiple. First, pectin participates in the self-assembly process during nanoparticle formation, providing an interphase between the hydrophobic core and the aqueous environment, regulating their size and aggregation. Second, the ratio between pectin and aleuritic acid is an important factor to consider. Low amounts of pectin slightly improve monomer condensation while higher amounts favor the inverse hydrolytic reaction. Third, self-assembly of *cutinsomes* from aleuritic acid with pectin on HOPG is affected by the interaction of the polysaccharide with the substrate, producing a structure of interconnected islands. On the other hand, the participation of pectin in the formation of *cutinsomes* from cutin monomers produces nanoparticles with lipid cores and diffuse shells composed by mixtures of lipids and the polysaccharide. Also, unlike the samples with aleuritic acid, pectin induce a gradual esterification of the cutin monomers as a function of the pH.

Despite extrapolation to an *in planta* scenario from the *in vitro* conditions above described is not straightforward, these results suggest that polysaccharides, specifically pectin, can interact favorably with cutin monomers, producing nanoparticles at neutral pH more esterified than those obtained polyhydroxylated fatty acid solutions. In other words, cell wall polysaccharides could play a significant role during the cutin biosynthesis beyond the mere mechanical support, participating actively in *cutinsome* formation.

## Supporting Information

S1 Fig TEM image of pectin stained with uranyl acetate. A blurry and slightly stained micrograph of pectin.
(TIF)
