# Supplementary figures and images for: Pectin-Lipid Self-Assembly: Influence on the Formation of Polyhydroxy Fatty Acids Nanoparticles
Source: PLoS One. 2015 Apr 27;10(4):e0124639. doi: 10.1371/journal.pone.0124639 (PMC4411075; doi:10.1371/journal.pone.0124639)

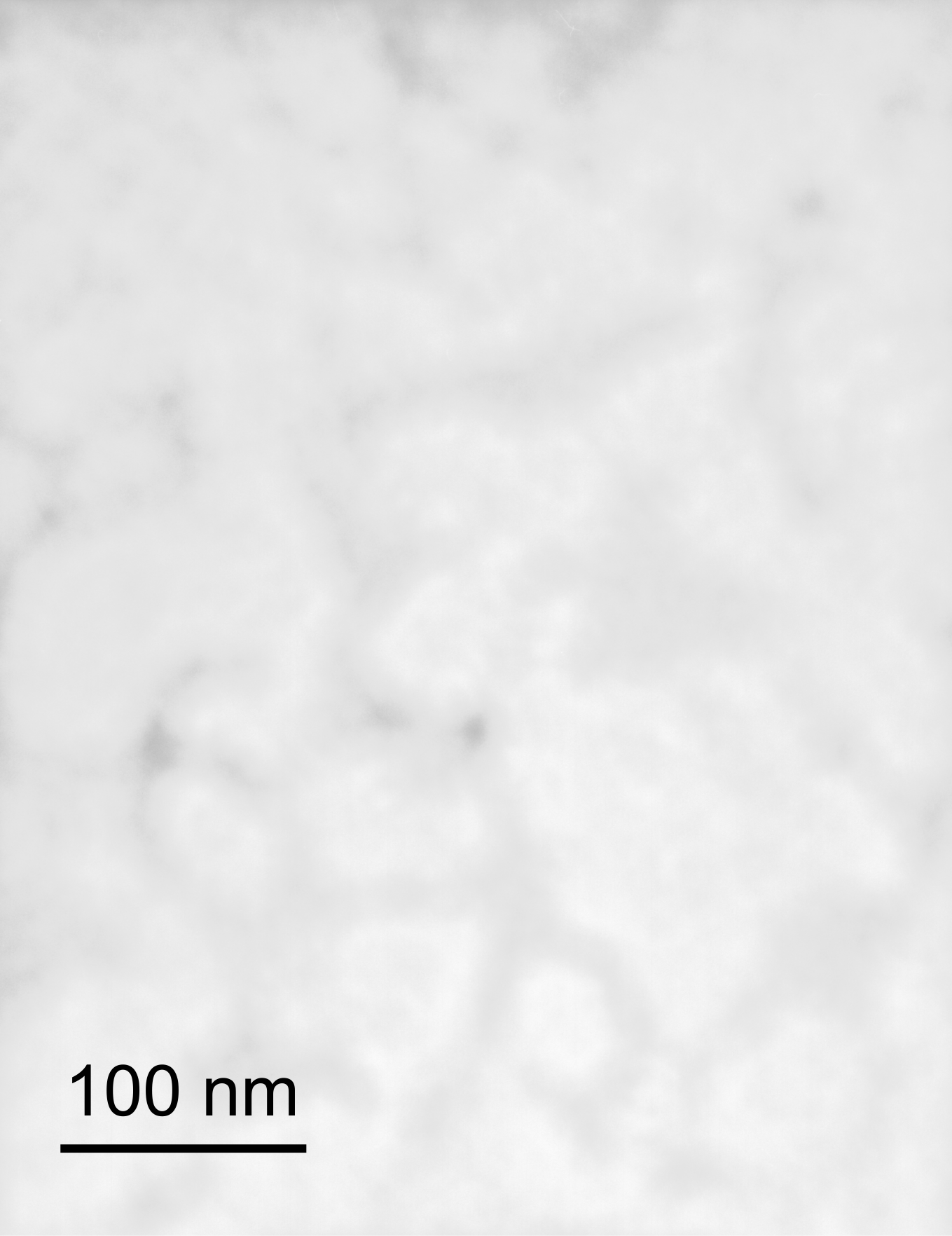

Supplement: S1 Fig — A blurry and slightly stained micrograph of pectin. (TIF) [file pone.0124639.s001.tif]
